# Suberin: the biopolyester at the frontier of plants

**DOI:** 10.3389/fchem.2015.00062

**Published:** 2015-10-30

**Authors:** José Graça

**Affiliations:** Centro de Estudos Florestais, Instituto Superior de Agronomia, Universidade de LisboaLisboa, Portugal

**Keywords:** suberin, suberized cell walls, poly(acylglycerol) macromolecule, ferulates of ω-hydroxyacids, cork, potato periderm

## Abstract

Suberin is a lipophilic macromolecule found in specialized plant cell walls, wherever insulation or protection toward the surroundings is needed. Suberized cells form the periderm, the tissue that envelops secondary stems as part of the bark, and develop as the sealing tissue after wounding or leaf abscission. Suberin is a complex polyester built from poly-functional long-chain fatty acids (suberin acids) and glycerol. The suberin acids composition of a number of plant tissues and species is now established, but how the polyester macromolecule is assembled within the suberized cell walls is not known. In the last years contributions from several areas have however significantly enriched our understanding of suberin. The primary structure of the polyester, i.e., how the suberin acids and glycerol are sequentially linked was revealed, together with the stereochemistry of the mid-chain functional groups some suberin acids have; solid-state NMR studies showed the presence of methylene chains spatially separated and with different molecular mobility; biophysical studies showed the membrane behavior of suberin acids derivatives, allowing new insights on structure-properties relationships; and a number of candidate genes were conclusively related to suberin biosynthesis. The comprehension of suberin as a macromolecule will be essential to understand its vital protective roles in plants and how they will deal with eventual environmental changes. Suberin is also expected to be a source for high-performing bio-based chemicals, taking advantage of the structural uniqueness of their constituent suberin acids.

## Suberized cell walls form a protective barrier in plants

When plants emerged from the sea and start evolving in the more aggressive land environment, they faced a number of survival challenges, including control of water loss, insulation against climatic variability, and protection against abiotic aggressions (Delaux et al., 2012). As an answer, plants developed tissues with barrier properties enveloping all parts of their body. Plant tissues of primary growth, like leaves, fruits, and stems, are covered by a single cell layer, the epidermis; in plants with secondary growth, like trees and shrubs, a multi-cell layer forms, the periderm, as part of the outer bark (Evert, 2006). To fulfill their frontier roles, epidermis and periderm cells have specialized biomacromolecules as a major component of their cell walls, cutin, and suberin, respectively.

Cutinized and suberized cell walls are however complex structures including, besides cutin and suberin, other biomacromolecules and non-polymeric components (the “extractives”). Polyaromatics, which can be analytically determined as lignin, are found associated (and covalently linked) to suberin, and in a lesser extent to cutin (Riley and Kolattukudy, 1975). Polysaccharides are also part of these cell walls, but in comparatively smaller proportions. Non-polymeric lipids, the “waxes,” are deposited in suberized and cutinized cells, sometimes in very significant amounts, and probably have a major role in the hydrophobicity and low permeability of these cell walls (Schreiber et al., 2005). Although cutin and suberin share many chemical and structural affinities, their building units (“monomers”) and macromolecular arrangement, follow different patterns in the respective cell walls. The focus of this mini-review is suberin, seen as a macromolecule in the context of the structure of suberized cell walls. Polyaromatics are briefly reviewed, taking into account their association with suberin; polysaccharides and non-polymeric lipids are not discussed, except on their putative roles in the overall structure of suberized cell walls.

**Figure 1:**
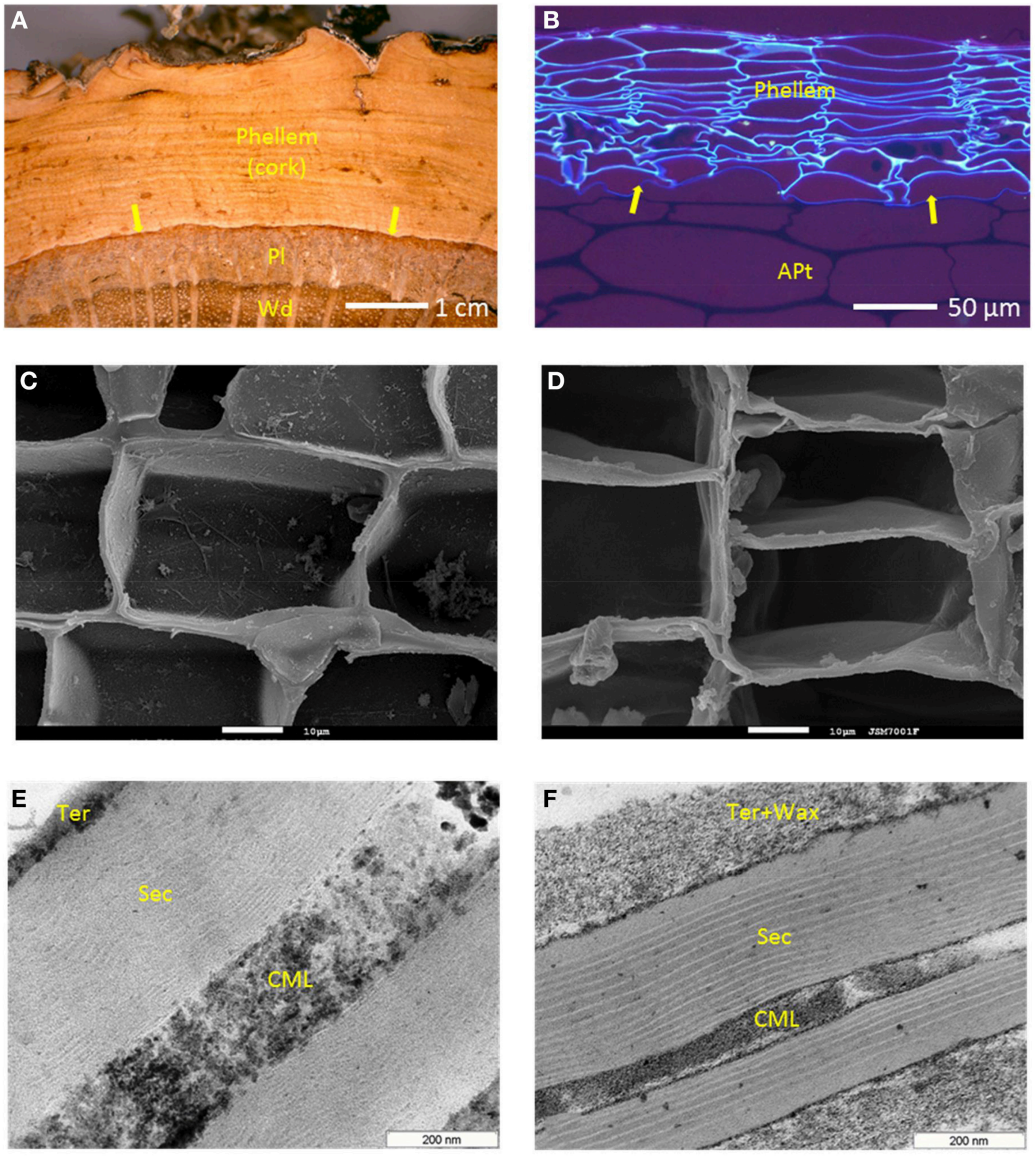
**Periderm and suberized cells in cork (***Quercus suber***) and potato tubers (***Solanum tuberosum***)**. **(A)** *Q. suber* trunk outer tissues, showing the periderm, made of phellem (suberized tissue, the *cork*), *phellogen* (the mother-cell layer, arrows), and phelloderm (not visible); *Pl*, phloem; *Wd*, xylem (wood). **(B)** Potato tuber outer tissues, showing the periderm, made of *phellem* (the suberized tissue), *phellogen* (arrows), and phelloderm (not visible); *Apt*, amylaceous parenchyma tissue. Suberized cells as seen by SEM, in cork **(C)** and potato periderm **(D)** (white bars = 10 μm). Ultrastructure of suberized cell walls as seen by TEM, in cork **(E)** and potato periderm **(F)**; *CML*, compound middle lamella (mostly primary wall); *Sec*, the poly-lamellate secondary wall; *Ter*, tertiary wall with the non-polymeric waxes, *Wax*, deposited.

Suberized cells are found not only as part of periderms, but also in other plant tissues, some of them internal, where control of water flow or transport to the surroundings is needed (Schreiber, 2010). Also, suberized cells form as wound tissue after physical injuries, as well as the sealing layer that leads to the abscission of plant parts (Vandoorn and Stead, 1997). However, it is in periderm of some plants that sometimes massive amounts of suberized cells are found. The periderm is formed by the phellogen, a meristematic cell layer that divides to the outside phellem, and to the inside phelloderm (Figures 1A,B). The phellem cells differentiate as suberized cells, and since the division of phelloderm is (in many cases) minimal, periderms are mostly made of suberized cells (Figures 1C,D).

In the context of trees biology, the suberized tissue of periderms is known as “cork.” Some trees and shrubs have a “corky” outer bark, because its phellogen is very active and divide large number of suberized cell layers each year of growth. Plants developed corky barks as an adaptation against fire, since they are found mostly in ecosystems prone to it, such as savannah and sub-tropical biomes (Dantas and Pausas, 2013). An exceptional corky outer bark is the one of the cork oak (*Quercus suber* L.), a tree found in the west Mediterranean and neighboring Atlantic regions, from which commercial cork is harvested. Cork have a large set of properties, making it a technological performing material, used worldwide in a high number of industrial applications, for some of them without proper substitute (Pereira, 2007). Many of these technical properties mimic the original functions of suberized cells in plants, and derive from the presence of suberin in their cell walls.

Periderms also form in underground stems, of which potato (*Solanum tuberosum*) tubers are a typical example. When potatoes are harvested, the suberization process keeps evolving in the periderm cells for a few days, making the “mature” potato skin (Lulai and Orr, 1994). The protection afforded by the periderm's suberized cells is essential for potatoes conservation, and the breaking of its integrity by physical or biotic damage a major problem in the respective agroindustry (Neubauer et al., 2013). When potato tubers are cut through, a sealing tissue made of suberized cells forms in a few days, making a “wound periderm” (Lulai and Corsini, 1998). Due to their abundance and industrial importance, the suberins of *Quercus suber* cork and potato periderm are the ones most extensively studied. Other aspects of the suberization process, like its physiology, genomics or suberin biosynthesis, were in many cases also carried out in potato wound periderms, due to the relatively short times and simplicity to get them.

## Suberized cell walls have a poly-lamellate organization

The specificities of suberized cell walls, in terms of their histology and structure, as observed at microscopic level, were early recognized. Still in the XIX century van Wisselingh observed that suberized cells walls showed three main layers, one more external corresponding to the primary wall, a thicker secondary wall faintly lamellate, and an internal tertiary wall (Vanwisselingh, 1888). The primary and tertiary cell wall layers were comparatively thin and stained positively for cellulose and lignin; the secondary wall was marked by lipid and fat stains and therefore included suberin in its composition. Some suberized cell walls also show deposited internally to the tertiary wall (or apparently in its substitution), other materials, which in some cases can represent a significant fraction of the wall's total thickness. These materials, the latter to be synthesized and deposited, are mainly extractable “waxes”: for instance, in potato periderm, this waxy layer in very significant (Figure 1F), accounting for 20% of the tissue weight (Graça and Pereira, 2000b). Suberized cell walls can be very thin: primary and tertiary walls have a thickness of 0.1 to 0.2 μm in cross section; the suberin secondary wall can have approximately 0.5 μm, and the extractives layer, when present, up to 0.3 μm (Figures 1E,F).

With the advent of the transmission electron microscopy (TEM), the pioneering work of Sitte (mostly based in cork) postulated in a comprehensive manner the ultrastructure of suberized cell walls. Besides confirming the overall structure proposed by van Wisselingh, the main observation of Sitte was that the secondary suberin wall is thinly lamellate (Sitte, 1959). Two types of alternating lamellae are observed: the “light,” electron translucent lamellae, and the “dark,” electron-opaque ones. In cork, Sitte measured for light lamellae an even thickness of 3 nm, and for the dark lamellae a variable thickness, from 7 to 10 nm. In all, from 30 to 60 light and dark lamellae could be counted across cork's suberized cell wall (Sitte, 1962). The number of observed lamellae is however highly variable depending on the particular tissue or plant species, and as rule, less in number than in *Q. suber* cork (Kolattukudy and Espelie, 1989). In some suberized cell walls the lamellae are very well-defined and straight, like in potato periderm (Figure 1E), but in others, they can look more undulated, particularly in the inner (lately differentiated) part of the secondary wall (Figure 1F).

The presence of this mostly organized poly-lamellate structure, besides tissue identity and location, has been used to define suberized cells (Kolattukudy and Espelie, 1989). In rare cases the lamellae were not observed in suberized walls (Teixeira and Pereira, 2010), but care should be taken since methodological choices can affect the visibility of the lamellae. The key question that remains to be answered is how the chemical composition of suberized cell walls is related to the observed ultrastructure; in particular, how the molecular (or supramolecular) arrangement of suberin, and other associated macromolecules, like polyaromatics, can explain the lamellate structure found in secondary walls. This will be addressed below together with the discussion of the macromolecular structure of suberin.

## Suberin is built from α,ω-bifunctional fatty acids and glycerol

**Figure 2:**
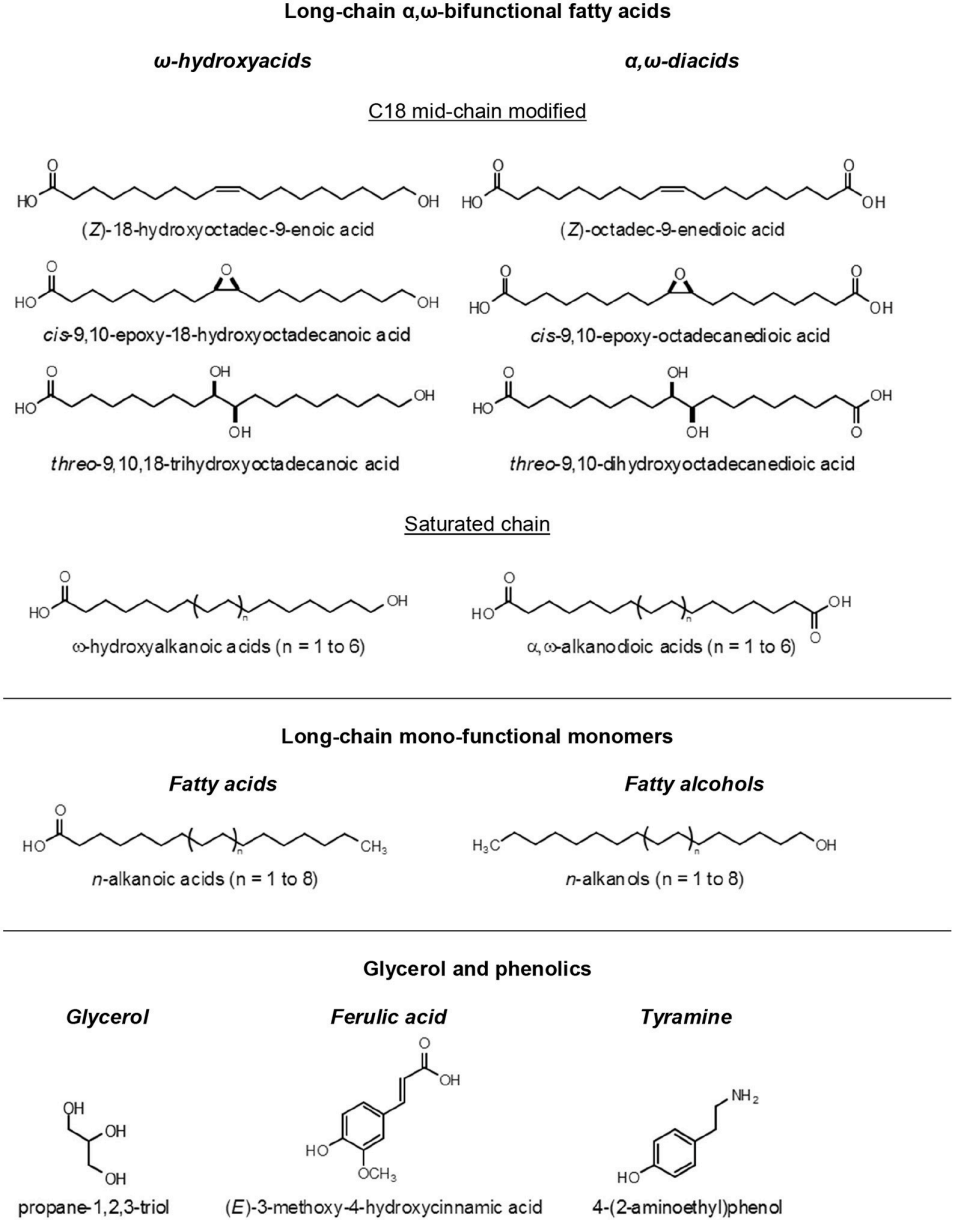
**Structural formula of the main suberin monomers**.

All types of ester breaking reactions can be used to depolymerize suberin, showing that its building units are interlinked as a polyester macromolecule. Alkaline hydrolysis was an initial favorite, but later, less aggressive techniques like base and acid-catalyzed methanolysis, and hydrogenolysis, have been preferred for analytical convenience (Kolattukudy, 2001). The major component of suberin depolymerisates are long-chain aliphatic acids, representing typically 80–90% in mass of all monomers released (Figure 2). Although a few ancient works had unequivocally showed that glycerol was also released when suberin was depolymerized (Ribas and Blasco, 1940), the presence of glycerol and its important role in suberin was ignored for a long time. Most of the suberin analysis put their focus on its aliphatic acids, which were recovered to an organic phase in partition with water, the latter carrying the glycerol away when discarded. Quantitative determinations of glycerol in suberins are relatively few, but values from 5 to 20% of all suberin monomers have been measured (Graça and Pereira, 2000a). Finally, when suberin is depolymerized, “phenolic acids” are also released; in particular ferulic acid is always present in small amounts. The role of this phenolic moieties in suberin is discussed in Section Polyaromatics are a Substantial Component of Suberized Cell Walls.

The total suberin content has been accessed as the organic soluble component of the depolymerization products (therefore not including glycerol) or more correctly, as the mass-loss after suberin depolymerization (Graça and Pereira, 2000a). Suberin content determinations in plant materials varied from 1% to more than 50%. However, besides the question of how the suberin content was calculated, these values are relative to plant materials which included very different proportions of suberized cells. In carefully isolated “pure” suberized tissue, after the removal of non-polymeric extractives, the suberin content is typically higher, from 10 to 60% (Holloway, 1983). This value should correspond to the proportion of suberin in the composition of suberized cell walls, and the one to take into account in the discussion in Section Suberin Make the Light, and Polyaromatics the Dark Lamellae in Suberized Cell walls.

The exact structure of suberin acids was long controversial, but by the middle of the XX century their chemistry was elucidated (Jensen, 1952; Ribas, 1952; Guillemonat, 1960). In the early 1970s, with the development of gas chromatography hyphenated with mass spectrometry (GC-MS), the analysis of complex mixtures of suberin acids become straightforward, and the works of Kolattukudy and Holloway pioneered the studies of suberin composition from a number of plant sources (Kolattukudy et al., 1975; Holloway, 1983). The main characteristic of suberin acids is that they are α,ω-bifunctional, i.e., they have linking groups at both ends of their hydrocarbon chains. Two main families of fatty acids are thus defined: the α,ω-diacids, with carboxylic acids at both the α and ω positions; and the ω-hydroxyacids, with a carboxylic acid in the α position and a hydroxyl group in the ω position (Figure 2). The existence of at least these two linking positions in suberin monomers is necessary for them to be part of a polymeric structure. Mono-functional fatty acids (alkanoic acids) and fatty alcohols (alkanols) are also commonly found among the depolymerization products of most suberins (Figure 2), but as a rule, they represent a comparatively small part of the long-chain monomers (< 10%); due to their single linking group they can only act as dead ends in the suberin macromolecule.

The chain length of both α,ω-diacids and ω-hydroxyacids in suberins range typically from C_16_ to C_26_, with the even numbered chains largely dominant (Holloway, 1983; Kolattukudy, 2002). In some suberins the mono-functional fatty acids and alcohols can reach much higher chain lengths, up to C_30_ (Rios et al., 2014). Within the α,ω-diacids and ω-hydroxyacids classes of suberin monomers, two groups are found: the C_18_ with mid-chain modifications (discussed below), and the ones with saturated chains, starting at C_16_ (skipping the C_18_) and going up to C_28_. Other types of fatty acids are also found in some suberins, including α-hydroxyacids, α,ω-alkanediols, and C_16_ ω-dihydroxyacids (Kolattukudy, 2002), the latter more typical of cutins.

The C_18_ mid-chain modified α,ω-diacids and ω-hydroxyacids are quantitatively relevant, or even dominant, in the composition of many suberins, and therefore must play a crucial role in its macromolecular structure. Three main types of mid-chain modifications are found at the C-9 and C-10 carbons: a double bond, an epoxide ring or two vicinal hydroxyl groups (*vic*-diol) (Figure 2). The stereochemistry of this mid-chain groups was studied in cork suberin: both the double bonds and the epoxide ring have a *cis* configuration, and the *vic*-diols are *threo*, using the nomenclature developed for carbohydrates for their relative configuration, meaning that both the *9R,10R*, and *9S,10S* absolute configurations can be present (Santos et al., 2013; Santos and Graça, 2014). These secondary groups at mid-chain can impact significantly the molecular packing of the suberin macromolecule due to steric effects, derived from their specific stereochemistry and volumetric hindrance, or provide cross-links though intra-molecular bonding (discussed below).

The relative proportion of the different families of monomers, and of the individual suberin acids, are highly variable in suberins, depending on plant species and tissue location (Holloway, 1983; Kolattukudy, 2002). The number of suberins which have been thoroughly analyzed so far is limited, but patterns in suberin composition can be recognized. As a rule, ω-hydroxyacids are always an important class of monomers; the α,ω-diacids can be second to ω-hydroxyacids, but can also be more abundant, as in the cases of *Q. suber* cork and potato periderm suberins (Graça and Pereira, 2000a,b). Two main types of suberins can be recognized taking into account their dominant suberin acids (Holloway, 1983): there is a group of suberins where the C_18_ epoxide and *vic*-diol α,ω-diacids and ω-hydroxyacids are overwhelming, with minor proportions of saturated chain monomers, like in *Kielmeyeria coriacea* bark periderm (Rios et al., 2014); and there is another group of suberins where the mono-unsaturated C_18:1_and saturated chain monomers are dominant, and the C_18_ epoxide and *vic*-diol suberin acids are present in small amounts or event absent, as are the cases in *Pseudotsuga menziessii* bark and potato periderm (Graça and Santos, 2007). This means that there are different patterns in the macromolecular structure of suberin. However, all kinds of in-between cases exist: for instance, in *Q. suber* cork, all types of the above monomers are found, and its suberin cannot be assigned to one of the groups (Graça and Santos, 2007). The reasons behind this variability are not clear, and can either be related to the ontogeny of the tissue, the physiological stress faced or genetics related to phylogeny.

## The primary structure of suberin: How are the suberin monomers sequentially linked?

How are the suberin monomers, namely the α,ω-bifunctional fatty acids, glycerol, and the phenolic acids inter-linked in the suberin macromolecule? So far our knowledge is limited to the so-called primary structure of the macromolecule, i.e., how the monomer residues are sequentially linked. Most of what is known comes from the analysis of oligomers, fragments of the suberin macromolecule still carrying some of their *in situ* inter-monomer ester linkages (Graça and Pereira, 1997; Wang et al., 2010). These oligomers (together with monomers) are obtained by the partial depolymerization of the suberin polyester, using mild or partial hydrolysis conditions. The structure of the solubilized oligomers is identified combining information from GC-MS, electrospray ionization coupled to tandem mass spectrometry (ESI-MS/MS) and high-resolution one and two-dimensional NMR. Oligomers including 2–5 monomer residues of all the main suberin monomers were found (Graça et al., 2015). Most of these results, discussed below, were obtained from *Q. suber* cork and potato periderm suberins.

The dominant oligomer species in the suberin partial depolymerisates are glycerides having as acyl moieties all types of suberin acids, namely α,ω-diacids, ω-hydroxyacids, and alkanoic acids (Graça and Pereira, 1999, 2000b). Acylglycerol species involving all the glycerol positions were found as suberin oligomers: 1(3)- and 2-monoacylglycerols, 1,2- and 2,3-diacylglycerols and triacylglycerols (Graça et al., 2015). Besides, α,ω-diacids were found esterified at both ends to two different glycerol moieties, with both 1(3)- and 2-glycerol positions involved in the ester linkages (Graça and Santos, 2006a). Oligomers showing the continuum of this glycerol—α,ω-diacid—glycerol structure units were also identified in the potato suberin partial depolymerisates (Graça et al., 2015). This glycerol—α,ω-diacid—glycerol block can therefore be one of the main backbones from which the suberin grows to macromolecular dimensions. In all, these results, together with the quantitative importance of the involved monomers, imply that suberin is structurally an acylglycerol lipid.

Besides linked to glycerol, suberin acids linked head-to-tail were also obtained as oligomer fragments from the partial depolymerization of suberins. These linear aliphatic esters included mainly as monomer residues ω-hydroxyacids, since the ω-hydroxyl group is necessary to make the ester bond; however, linear ester oligomers including α,ω-diacids were also detected (Graça and Santos, 2006b). The number of suberin acids included in these linear esters were no more than three, as found in the partial depolymerisates of the analyzed suberins (Graça et al., 2015). Some of these rows of interesterified suberin acids were further linked at their end to glycerol. This means that the suberin macromolecule also grows linearly, by the successive addition of ω-hydroxyacids at end chain.

The other significant ester structure found in the oligomers released from the partial depolymerisation of suberins are ferulates of ω-hydroxyacids. In the suberins analyzed so far (*Q. suber* cork, *P. menziesii* bark cork and potato periderm), all ω-hydroxyacids which were found as monomers, were also found esterified though their ω-hydroxyl group to ferulic acid as oligomer species (Graça and Pereira, 1998, 1999, 2000b). Also, the trimeric structure of glycerol esterified to ferulates of ω-hydroxyacids, through the acidic end group of the latter, was found in the partial depolymerisates of suberins (Santos and Graça, 2006; Graça et al., 2015). This means that one of the main roles of the ω-hydroxyl group of ω-hydroxyacids is to link the acylglycerol matrix of suberin to phenolic moieties. Due to the high proportion of ω-hydroxyacids found as suberin monomers, and the relative abundance of their ferulates found as suberin oligomers, these aliphatic-aromatic linkages can be an important feature in the organization of suberized cell walls. The discussion of the association of the suberin polyester with aromatics follows.

## Polyaromatics are a substantial component of suberized cell walls

Suberized cell walls include non-soluble polyphenolic materials, the “polyaromatics,” in significant quantities: in cork, assessed as lignin, they represent approximately 25% of all structural components (“extractive-free” basis) (Pereira, 1988); in native potato periderm, 31% of insoluble phenolics were determined based in the initial weight, after suberin, polysaccharides and extractives removal (Mattinen et al., 2009); even higher values have been found in wound-induced potato periderm, with a ratio of polymeric phenolics to aliphatic suberin of 2:1, as measured by the respective carbon peak areas in solid-state ^13^C NMR analysis (Garbow et al., 1989); in the periderm of six subterranean periderms, residual polyaromatics, after the removal of ester-linked suberin, represented from 25 to 30% of the extractive-free initial material (Kolattukudy et al., 1975). A differential scanning calorimetry (DSC) analysis of a potato periderm extract enriched in suberin and polyaromatics showed two different polymer softening temperatures, at 45°C and 59°C, with the latter eventually arising from the polyaromatics moiety (Mattinen et al., 2009). What exactly these polyaromatics are and how similar they are to conventional lignins, remains controversial.

Besides the insoluble polyaromatics, when the suberin polyester is depolymerized by any ester breaking technique, small amounts of phenolics are co-solubilized together with the main aliphatic suberin monomers. Values from 1 to 10% of all suberin depolymerized materials have been found (Kolattukudy et al., 1975; Borgolivier and Monties, 1993). Among the soluble phenolics detected, the hydroxycinnamic ferulic acid is always present in comparatively higher quantities (Figure 2), and can have a significant role in the overall structure of suberized cell walls (Graça, 2010). Aminated phenolics have also been found co-solubilized from suberin, namely tyramine (Figure 2), both in native and wound induced potato periderm, however in quantities even smaller than ferulic acid (Borgolivier and Monties, 1993). Also, in potato periderms, two ferulic acid amides, feruloyltyramine, and feruloyloctopamine, were found ether linked in the insoluble polyaromatics, but there was no proof of their direct linkage to suberin (Negrel et al., 1996).

The analysis of the insoluble suberin-associated polyaromatics have included, besides degradative lignin techniques, like thioacidolysis and analytical pyrolysis coupled to mass spectrometry (Py-GC-MS), also *in situ* studies by ^13^C solid state NMR (^13^C ssNMR). A model for the suberin polyaromatics as a poly(ferulic acid) structure, built from ferulic acid units linked by condensation and ether linkages similar to lignin, was developed: after feeding ^13^C labeled phenylalanine in suberizing wound potato periderm, signal enhancement was found mostly in carbons assignable to hydroxycinnamates (Bernards et al., 1995; Bernards and Lewis, 1998). This ferulic-acid based polymer was interpreted as a “non-lignin” biomacromolecule (Bernards and Razem, 2001). However, a number of results showed that the suberin-associated polyaromatics behaved analytically (at least in part) as typical lignins. Work carried out in the cork periderms of a few trees and also in native potato periderm, showed these polyaromatics to be dominantly a guaiacyl lignin (Neto et al., 1996; Mattinen et al., 2009; Marques and Pereira, 2013), although in wound potato periderm comparatively high proportions of syringyl units were also found (Lapierre et al., 1996; Yan and Stark, 2000). The exact structure and homogeneity of the suberin-associated polyaromatics remains an open question.

## Suberin is a poly(acylglycerol) macromolecule in part arranged as a lipid membrane

A key question to be answered is how we define suberin. The original term “subérine” (in French) is attributed to the famed XIX century chemist Chevreul, who used it to define all the non-soluble materials specific of cork (Gilson, 1890). Later, suberin was considered as the component susceptible to be depolymerized by ester-breaking treatments, thus corresponding to the aliphatic polyester. In a more recent interpretation, suberin has being used to name the two main components of suberized cell walls, thus including two “domains”: the aliphatic polyester named the “suberin poly(aliphatic) domain” (SPAD), and the insoluble polyaromatics, named the “suberin poly(phenolic) domain” (SPPD) (Kolattukudy et al., 1975; Bernards, 2002). In our point of view, the name suberin should be kept for the aliphatic polyester (as followed in this review) for several reasons: first, the aliphatic suberin can be analytically isolated as such; second, the polyaromatics are chemically and structurally distinct and in part, spatially separated in suberized cell walls; and finally, the suberin aliphatic polyester can have a defined macromolecular and independent structure (as discussed below). This doesn't preclude the existence of extensive contact and covalent linkages between suberin and the associated polyaromatics, such as those known between other biomacromolecules in plant cell walls.

**Figure 3:**
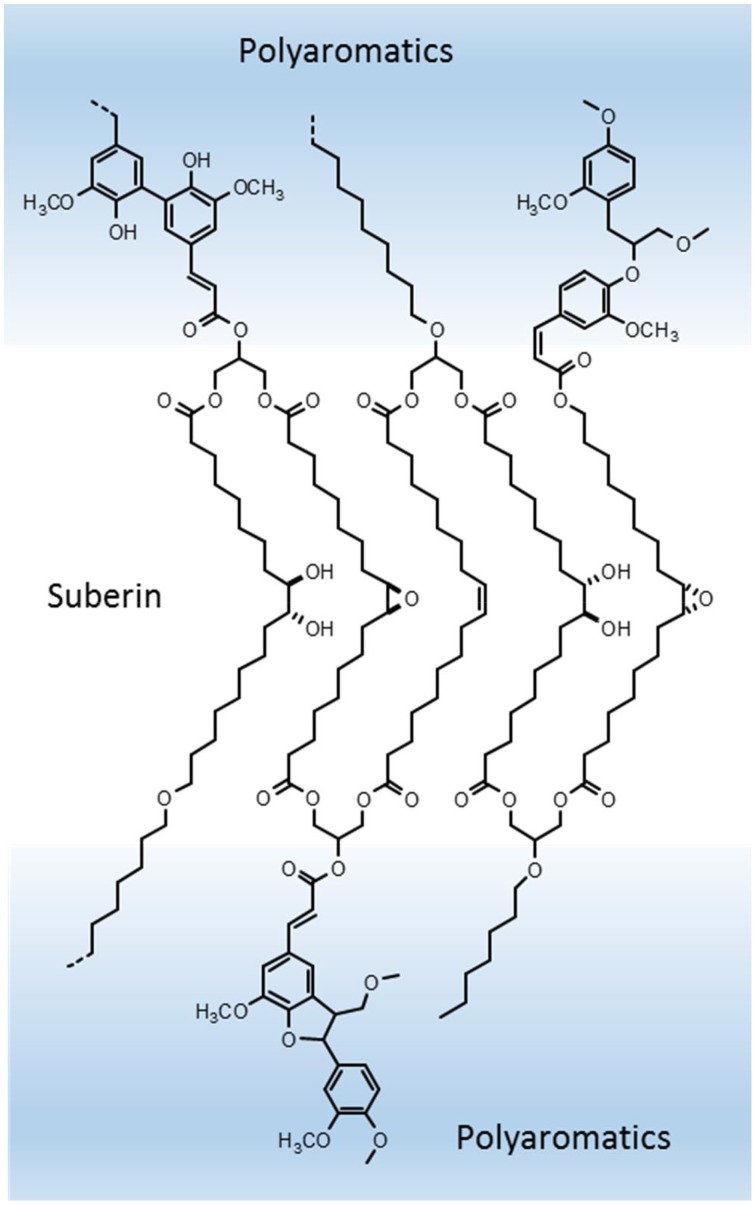
**Suberin hypothetical model in the context of suberized cell walls structure: core suberin, a regularly packed poly(acylglycerol) macromolecule, makes the translucent lamellae; this suberin polyester is covalently linked through esterification to ferulic acid to the neighboring lignin-like polyaromatics, which account for the dark lamellae**.

Based in the above assumptions and in our present knowledge, suberin can be defined as a poly(acylglycerol) macromolecule. Glycerol units linked in succession to α,ω-diacids will be the core backbone of the suberin macromolecule (as illustrated in Figure 3); this successive glycerol—α,ω-diacid—glycerol connections can grow as a reticulated and disordered net, giving rise to a random structure (Pollard et al., 2008), or the hydrocarbon chains can dispose in a more orderly packed manner, building membrane-like regions within the suberin macromolecule (Graça and Santos, 2007). Some other indirect evidence can be seen as supportive of the above proposed structure. Suberin-like glycerol—α,ω-diacid—glycerol model molecules were shown to self-assembly *in vitro*: synthesized glycerol—1,16-hexadecanedioic acid—glycerol formed stable lipid vesicles in aqueous media; these vesicles became fluid only at relatively high temperatures (57°C), and their membrane thickness could be estimated close to the length of the C_16_ chain when stretched (Douliez et al., 2005). ^13^C ssNMR molecular dynamics studies in both cork and potato periderm suberins revealed the presence of two different populations of aliphatic CH_2_ (methylene) groups, one more mobile, and the other, presumably close to ester groups (as shown by their chemical shifts), much more rigid in molecular motion (Gil et al., 1997; Yan and Stark, 1998); relating these results to the acylglycerol structure proposed here for suberin, the rigid methylene moieties can correspond to the hydrocarbon chains in the more orderly organized parts of the suberin polyester, and the ones with more motional freedom to the hydrocarbon chains in less constrained regions, further apart from the glycerol anchorage points.

Mid-chain modifications in the C_18_ ω-hydroxyacids and α,ω-diacids, dominant in many suberins, can add further complexity to its macromolecular structure and affect the overall properties of suberized cell walls. Unsaturated C_18:1_suberin monomers, due to its *cis*-configuration, can bend the hydrocarbon chain to a kinked position; fatty acids with mid-chain *cis*-double bonds are known to impart fluidity to lipid membranes, which could be the case in the suberins where these C_18:1_suberin are dominant. In the opposite direction, the C_18_ 9,10-epoxide and 9,10-*vic*-diol substituted monomers can eventually carry rigidity to the structure: they can also bend at mid-chain, favoring the establishment of intra-monomer secondary bonds, namely hydrogen bridges, cross-linking the hydrocarbon chains, and thus reinforcing an eventual packed arrangement of the suberin acylglycerol structure (Figure 3).

As discussed above, we know that ω-hydroxyacids (at their ω-hydroxyl end) show up commonly in suberin oligomers as ferulates. This means that ω-hydroxyacids, besides being part of the acylglycerol suberin matrix, linked through their acidic end group to glycerol, can act at the same time as connectors to the neighboring polyaromatics, through esterification to ferulic acid; the latter can be part of, or be linked to the periphery of those polyaromatics (Figure 3). We also know that ω-hydroxyacids are ester linked head-to-tail as linear structures. How long this rows of long-chain monomers grow, we don't know. They can eventually be part of a continuum of the aliphatic polyester across the suberized cell walls. As mentioned, since there are suberins with different proportions of ω-hydroxyacids, α,ω-diacids and glycerol, together with different monomer compositions, the existence of variable suberin macromolecular arrangements are to be expected.

## Suberin make the light, and polyaromatics the dark lamellae in suberized cell walls

How does the proposed model for the suberin polyester and associated polyaromatics can fit into the ultrastructure of suberized cell walls as observed by TEM? We know that the suberized cell walls have a lamellate structure, in which the light-contrast lamellae have a regular thickness, and the dark-contrast lamellae a not so regular thickness. Initially, the light lamellae were attributed to non-polymeric waxes and the dark lamellae to suberin (Sitte, 1962). This idea was supported by the observation that in wound potato periderm, the light lamellae did not form after the specific inhibition of waxes synthesis (its chain elongation impaired by trichloroacetate), without affecting significantly suberin monomer composition (Soliday et al., 1979). But other results are consistent with the location of the suberin aliphatic polyester in the light lamellae. In the suberized fibers of cotton, the application *in vitro* of a specific inhibitor of fatty acid elongases (EPTC), led to a suberin with shorter long-chain monomers; besides, the suberized cell walls after this treatment showed less, and in some cases thinner, light lamellae; the conclusion was that the glycerol-suberin acids structure was necessary for the lamellae formation, and that the long-chain monomers would be perpendicular to the light lamellae plane (Schmutz et al., 1996). Also in potato periderm, the silencing of a key gene involved in the ω-hydroxylation of fatty acids in suberin, *CYP86A33*, led to much less suberin, highly deficient in C_18_ ω-hydroxicadis and α,ω-diacids; as a result, the lamellar structure was disrupted (Serra et al., 2009).

The analysis of suberized cell walls *in situ* by ^13^C ssNMR techniques allowed direct information on the location and molecular mobility of their aliphatic and aromatic components, both in potato periderm (Stark and Garbow, 1992; Yan and Stark, 1998) and in cork (Neto et al., 1995; Gil et al., 1999). The aliphatic polyester is spatially separated from the polyaromatics, and the latter have two populations, one in the vicinity of the aliphatic polyester and the other close to polysaccharides. Considering all the available information above discussed, we can hypothesize the following role of suberin as part of the topochemical arrangement of suberized cell walls: light lamellae are made by the core of the suberin polyester, namely glycerol—C18 α,ω-diacids—glycerol and the glycerol—C18 ω-hydroxyacids moieties; in this suberin regions, hydrocarbon long-chains will be in part orderly arranged, accounting for the regular thickness of the light lamellae; the dark lamellae are mostly made by the polyaromatics, though locally crossed by aliphatic chains; suberin and the vicinal polyaromatics are extensively covalently linked at their interface through ferulic acid, the latter esterified to ω-hydroxyacids and glycerol on the polyester side, and with lignin-like linkages on the polyaromatics side; this polyaromatics are a ferulic-acid rich lignin-like macromolecule; the polyaromatics not vicinal to the suberin polyester are a standard, dominantly guaiacyl lignin, that together with polysaccharides, build the primary wall (and tertiary wall when present); inner to the suberin cell wall, non-polymeric materials, namely “waxes,” are deposited as a more or less thick layer, depending on their relative abundance (Figure 2).

## Genomics and biosynthesis studies go together with the suberin chemistry structural results

Studies on the biosynthetic pathways leading to suberin (and cutin) monomers started in the 1970s (Kolattukudy and Espelie, 1989), but most of the knowledge on the specific genes and proteins involved on the monomers synthesis and their assembling as a polyester in suberizing plant cells was obtained in the last decade. Biosynthesis studies were done mostly in suberizing wound potato, but also in other plants and tissues where cutin is formed, since they share a number of monomers (Kolattukudy, 2002). Many of the genomics and associated biochemical and functional studies were carried out in the model *Arabidopsis*, namely through silencing and/or overexpressing of suberin candidate genes, and assessing the phenotypical modifications at the polyester structure and composition (Beisson et al., 2012; Franke et al., 2012). Biosynthesis studies showed that the mono-functional 9-unsaturated C_18:1_(oleic) acid was the starting point for the mid-chain 9,10 modified suberin monomers: the terminal C-18 carbon is first ω-hydroxylated forming the C_18:1_ω-hydroxyacid, and further oxidized to the corresponding C_18:1_α,ω-diacid; the epoxidation at the C-9/C-10 carbons, and its further hydration, give rise respectively to the C_18_ 9,10 epoxide and C_18_9,10-diol monomers. Straight chain suberin acids followed the known pathway starting at acetyl and malonyl CoA, down to the C_16_ saturated chain fatty acid, followed by its successive elongation, together with its eventual end chain oxidation to ω-hydroxyacids and α,ω-diacids (Kolattukudy, 2002).

Recent genomics approaches allowed the identification of enzymes, and their encoding genes, that lead not only to suberin acids, but also to the different ester structures found in suberin known from the chemical analysis. Cytochrome P450(CYP) oxygenases were proved to play a crucial role in the end- and mid-chain oxidation steps, starting from mono-functional fatty acids, and leading to ω-hydroxyacids and α,ω-diacids, either with saturated chains or with secondary oxygenated groups (Pinot and Beisson, 2011). Some genes and the corresponding enzymes seem to be selective for specific suberin monomers: genes encoding ω-hydroxylases more specific to C_16_–C_18_ (*CYP86A1*) or to C_22_–C_24_ fatty acids (*CYP86B1*), were found associated with the suberization process; the epoxidation *in vitro* of the C-9 double bond of the C_18:1_(oleic) acid by the CYP94A1 oxygenase, gave rise to dominantly (*9R,10S*) and much less (*9S,10R*) stereoisomers, both corresponding to the *cis* configuration found in suberin epoxyacids discussed above. The silencing of the *CYP83A33* gene in potato, which promotes the ω-hydroxylation step in its periderm suberin, led to a highly modified polyester structure and the distortion of the lamellar arrangement in the cell walls (Serra et al., 2009).

Acyltransferases, and the corresponding genes, able to synthesize two of the main ester structures found in suberin have been identified, promoting the esterification of suberin acids to glycerol, and the esterification of ω-hydroxyacids (through their ω-hydroxyl group) to ferulic acid. A glycerol-3-phosphate acyltransferase (GPAT5) was found to direct the esterification of ω-hydroxyacids and α,ω-diacids at the *sn*-2 position (the “middle” hydroxyl) of glycerol (Yang et al., 2010), and other GPATs are expected to carry the esterification at the *sn*-1(3) positions (Beisson et al., 2012). Feruloyl transferases from the BAHD family able to ester-link ferulic acid to ω-hydroxyacids (and *n*-alkanols) were found in suberizing tissues, and their role in the suberin biosynthesis proved after their silencing in mutant plants (Molina et al., 2009); these ω-hydroxyacid ferulate connections were discussed above as the putative preferential structure that links the suberin aliphatic polyester to the neighboring aromatics. In spite of these significant advances, how the suberin monomers assemble *in situ* building the macromolecular structure, and the transport processes involved, remains in most part to be elucidated.

## The chemical singularity of suberin makes it valuable for industrial applications

The chemical uniqueness of suberin long-chain α,ω-bifunctional monomers, or the polyester macromolecule as a whole, makes them of high industrial interest. A key point is their bi- or poly-functionality: each suberin acid have at least two reactive groups in the α,ω-positions, or more in the case of the C_18_ mid-chain modified ones; besides, they carry the properties derived from the long hydrocarbon chains, such as molecular flexibility and hydrophobicity. The preparation of technical polymers with designed properties, starting from these bi- or polyfunctional suberin monomers, is one of the more promising areas (Olsson et al., 2007; De Geus et al., 2010; Sousa et al., 2011). Because of this, considerable efforts have been done to obtain suberin-like fatty acids by biotechnological means (Huf et al., 2011) and chemical synthesis (Yokota and Watanabe, 1990). However, suberin-rich plant materials are available as residues in thousands of tons from some industries, like cork transformation (cork powders), potato processing (potato skins) or birch (*Betula* sp.) trees harvesting (outer bark) (Pinto et al., 2009). In the perspective of its industrial use, the extraction of suberin from the suberized matrices has been made by conventional hydrolytic means, and more recently, was achieved using ionic liquids, specifically targeting the acylglycerol linkages (Ferreira et al., 2014).

The efforts to obtain isolated suberin acids in industrial scale date back to the 1940s when a company named “Suber” was installed in France, offering “Subéryl,” a product obtained from suberin after cork saponification (Guillemonat, 1949). At research level, the number of assayed applications and potential uses for suberin and suberin acids keeps growing: hybrid co-polymers like polyurethanes were made from cork suberin extracts and isocyanate monomers (Cordeiro et al., 1999), and thermoset resins from epoxy ω-hydroxyacids and methacrylates (Torron et al., 2014); polymers built from the polymerization of long-chain ω-hydroxyacids were used to obtain high-resistant fibers (De Geus et al., 2010); and suberin fatty acids were shown to improve significantly the water vapor imperviousness of cellulose-based films (Heinäemäeki et al., 2015). Moreover, suberin seems to be of potential interest in many other areas. For instance, suberin extracts showed cancer-preventing anti-mutagenic properties (Krizkova et al., 1999) and a firming anti-wrinkle action in human skin (Coquet et al., 2005). Suberin has also been regarded as an inspiring source for biomimetic materials, including “superhydrophilic” and “superhydrophobic” surfaces (Koch and Barthlott, 2009). Finally, another major asset of these suberin-based products is that they can be obtained from renewable and sustainable plant sources, thus insuring its future development.

### Conflict of interest statement

The author declares that the research was conducted in the absence of any commercial or financial relationships that could be construed as a potential conflict of interest.
